# Evaluation of the osteogenesis and osseointegration of titanium alloys coated with graphene: an *in vivo* study

**DOI:** 10.1038/s41598-018-19742-y

**Published:** 2018-01-30

**Authors:** Kewen Li, Chunhui Wang, Jinhong Yan, Qi Zhang, Baoping Dang, Zhuo Wang, Yun Yao, Kaifeng Lin, Zhongshang Guo, Long Bi, Yisheng Han

**Affiliations:** 1 Department of Orthopedics, Xijing Hospital, The Fourth Military Medical University, Xi’an, 710032 P.R. China; 2 Department of Orthopedics, Qinghai University Affiliated Hospital, Xining, 810001 P.R. China; 3 Military Frontier Defence Medical Service Tranning Group, Army Medical University, Hutubi, Xinjiang 831200 P.R. China

## Abstract

The aim of this study was to investigate whether a surface coating with graphene could enhance the surface bioactivation of titanium alloys (Ti_6_Al_4_V) to further accelerate *in vivo* osteogenesis and osseointegration at the implant surface. In this study, a New Zealand white rabbit femoral condyle defect model was established. After 4, 12 and 24 weeks, biomechanical testing, micro-computed tomography (Micro-CT) analyses and histological observations were performed. At the highest push-out forces during the test, microstructure parameters, such as the bone volume/total volume fraction (BV/TV) and mineral apposition rate (MAR), of the new bone were significantly higher in the graphene-coated Ti_6_Al_4_V group (G-Ti_6_Al_4_V) than in the Ti_6_Al_4_V group (*P* < 0.05). Van Gieson (VG) staining showed that the G-Ti_6_Al_4_V group had more new bone formation than the Ti_6_Al_4_V group, and the G-Ti_6_Al_4_V group showed a closer fit between the bone and implant. In conclusion, graphene might be a novel type of nano-coating material for enhancing the surface biological activity of Ti-based alloy materials and may further promote *in vivo* osteogenesis and osseointegration.

## Introduction

Titanium alloys are one of the most important biomedical metals widely used in orthopedic implants due to their high mechanical strength, fracture toughness and corrosion resistance and their excellent biocompatibility^1–3^ as artificial bones and joints, as well as plates, screws and substitute materials for other hard tissues^4–9^. Although titanium alloys have been used in clinics for more than 30 years, there are still flaws that need to be resolved. In addition to mechanical and biological properties, the key to a successful implant is the initial fixation strength and long-term osteointegration of the implant/bone interface. Since titanium alloys are very readily oxidized, a layer of dense and strong TiO_2_ film develops on the surface of titanium alloys, which provides an underlying substrate with strong corrosion resistance. This property makes titanium and titanium alloys attractive for use in implants^10^, but the oxidized layer of titanium alloys is a biologically inert film that hinders the direct interaction of the implant with bone tissues^11^, resulting in Ti-based implants that may fail due to insufficient integration into the surrounding bone. To address this issue, coating implant surfaces with bioactive substances has been shown to effectively improve the biocompatibility and bioactivity of implants, promote bone tissue regeneration, and improve bonding strength and osteointegration at the implant/bone interface^12^.

Graphene is a two-dimensional atomic crystal comprising a single atomic layer formed by sp^2^ hybridization. The material has unique optical, mechanical, chemical and electrical properties^13–15^ and has been extensively studied by materials scientists, physicists and chemists^16,17^, and its applications span various fields, from electronics and chemistry to biomedicine^18^. Graphene performs well in medical applications, such as for cancer treatments^19,20^, as a drug carrier^21,22^, as a biosensor^23,24^, in biological imaging^25,26^ and as a biological scaffold in tissue engineering^27,28^. Previous studies have revealed that the use of graphene as a biological scaffold can enhance cell differentiation^29–31^. Kim *et al*.^32^, discovered that the special nanomorphology of graphene (asymmetric nanostructures) and its secondary properties, such as the rigidity and roughness of a graphene layer, play an important role in promoting the osteogenic differentiation of human mesenchymal stem cells (HMSCs). In this study, we prepared graphene coatings (G) on the surface of the Ti_6_Al_4_V and performed *in vivo* experiments. We hypothesize that the outstanding surface bioactivity and electrical property of graphene should stimulate the osteogenic differentiation into osteogenic lineages, thus improving the initial fixation strength and long-term osteointegration of the implant/bone interface to further promote bone defect healing.

## Results

### Characterizations of graphene coatings

**Figure 1:**
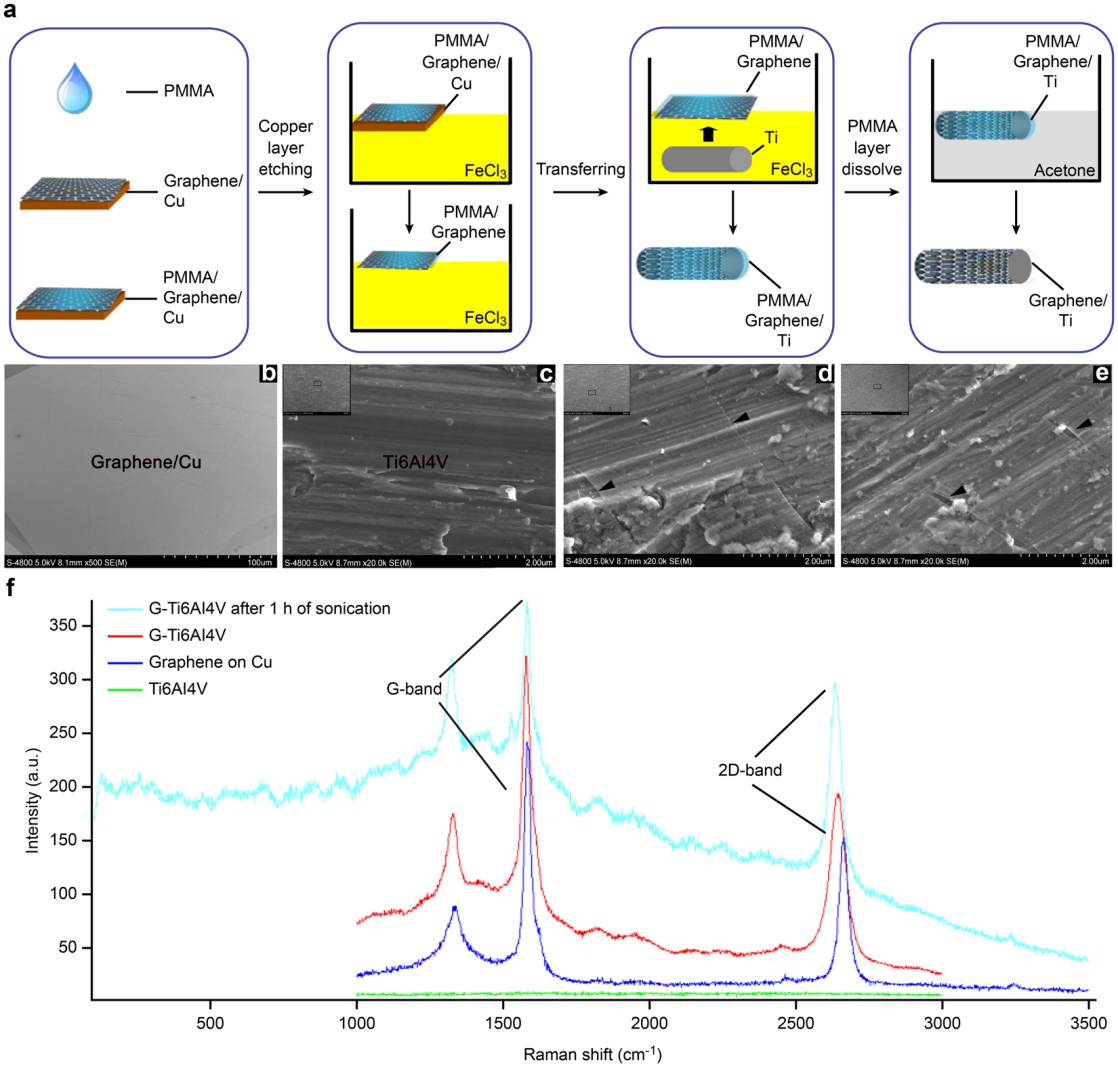
(**a**) Schematic diagram of the processes used for the surface etching and transfer of graphene to a copper substrate and the modification of the scaffold. (**b**) G-Cu: the entire surface is covered with a layer of gray graphene film (n = 5, graphene coverage of 100%). (**c**) SEM Ti_6_Al_4_V image: only scratches ae visible on the surface after polishing (n = 5). (**d**) G-Ti_6_Al_4_V: the black arrows point to the graphene film and wrinkles (n = 5). (**e**) G-Ti_6_Al_4_V: after 1 h of sonication, the graphene coating (black arrows) was still stable (n = 5). (**f**) Raman spectroscopy (laser wavelength = 630 nm): the nattier blue peak corresponds to G-Ti_6_Al_4_V after 1 h of sonication, the red peak corresponds to G-Ti_6_Al_4_V, the deep blue peak corresponds to G-Cu, and the characteristic graphene G peak (1,580 cm^−1^) and 2D peak (2,660 cm^−1^) are clearly present. The green peak corresponds to the uncoated Ti_6_Al_4_V, which does not show the two characteristic graphene peaks in the Raman spectra (n = 5).

Figure 1a shows a schematic diagram of the corrosion and adsorption methods for coating graphene onto a Ti_6_Al_4_V surface. Scanning electron microscopy (SEM) observations were obtained after coating and ultrasonicating. As shown in Fig. 1b, the entire surface is covered by a layer of gray film. Figure 1c shows scratches on the surface of the titanium alloy after polishing, and a film is not visible. Figure 1d shows scratches on the Ti_6_Al_4_V surface after polishing, but wrinkles in the film (black arrows) are also visible. After 1 h of sonication, the G-Ti_6_Al_4_V still showed a thin film (Fig. 1e, black arrows), and Raman spectroscopy was used to determine whether this thin film was graphene. Figure 1f shows the results for five samples each for G-Cu (deep blue peaks), G-Ti_6_Al_4_V (red peaks), and G-Ti_6_Al_4_V (nattier blue) after 1 h of sonication. We randomly selected six sites on the surface for Raman spectroscopic detection and observed more obvious characteristic graphene peaks (G and 2D). For the uncoated Ti_6_Al_4_V (green) samples, G and 2D peaks were not observed, indicating that the film observed in the SEM image is graphene, as well as suggesting that the titanium alloy was successfully coated and that the coating is stable.

### Biomechanical testing

**Figure 2:**
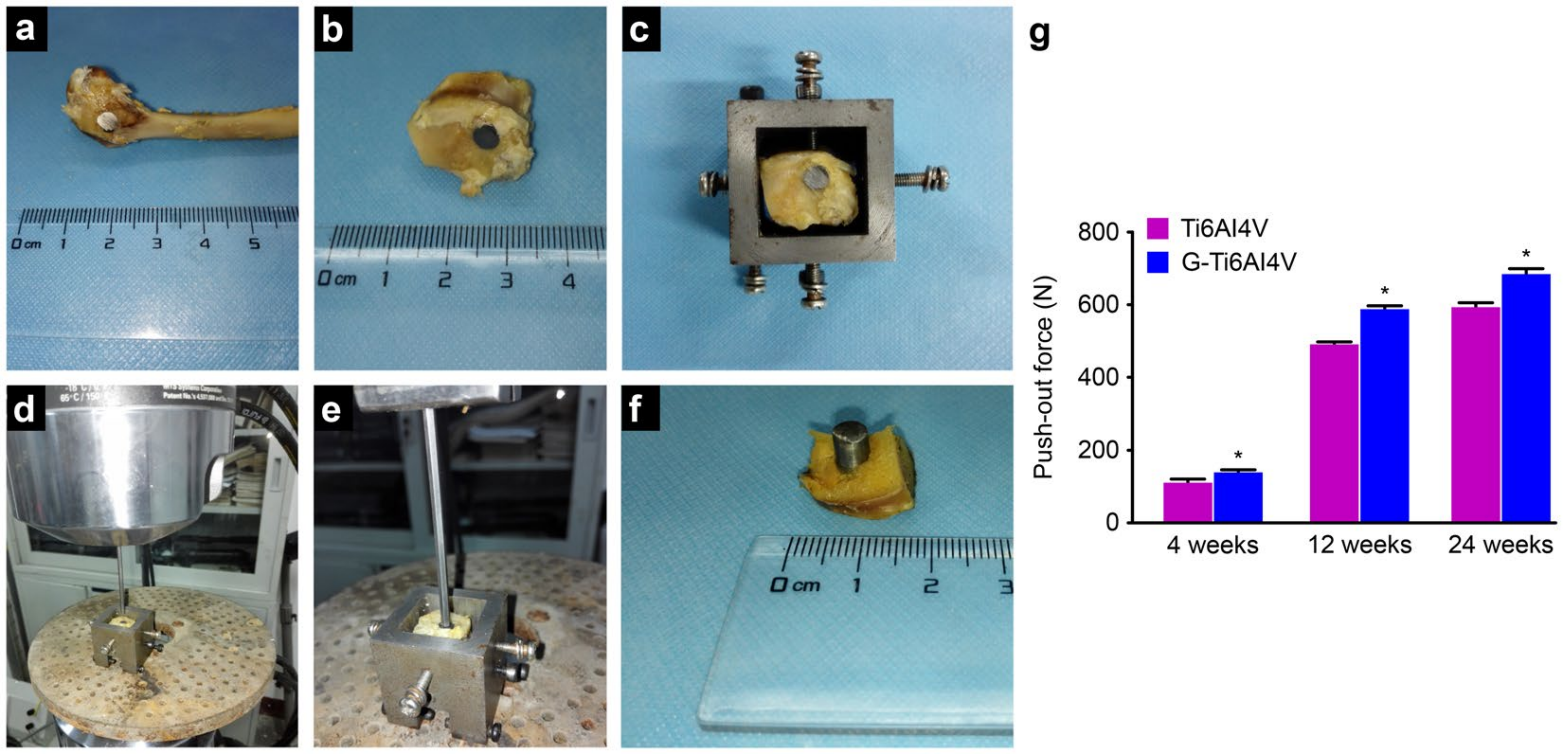
(**a**) Completely removed femoral condyle. (**b**,**c**) After trimming, the condyle was placed into a special fixture. (**d**,**e**,**f**) Push-out test: the load speed was set to 1 mm/min, and the maximum failure load was recorded. (**g**) Maximum failure load of the two groups after 4, 12 and 24 weeks (n = 6). The results are expressed as the mean ± SD; *represents a significant difference (*P* < 0.05).

The femoral condyles of each specimen were extracted 4, 12 and 24 weeks after scaffold implantation (Fig. 2a) and trimmed to fit the size of the special fixture of the testing machine (Fig. 2b and c). Metal rods (*Φ* = 4 mm) at a loading speed of 1 mm/min were used to force the implants to gradually detach from the femoral condyle (Fig. 2d,e and f). As shown in the Fig. 2g, the maximum failure load of both groups increased over time. However, at each time point, the maximum failure load of the G-Ti_6_Al_4_V group was significantly greater than that of the Ti_6_Al_4_V group (*P* < 0.05), indicating that detachment from the condyle required a greater push-out strength for the graphene-coated group than for the non-coated group. These results show that the strength of binding between the scaffold and the bone interface can be enhanced by a graphene coating.

### Micro-CT detection

**Figure 3:**
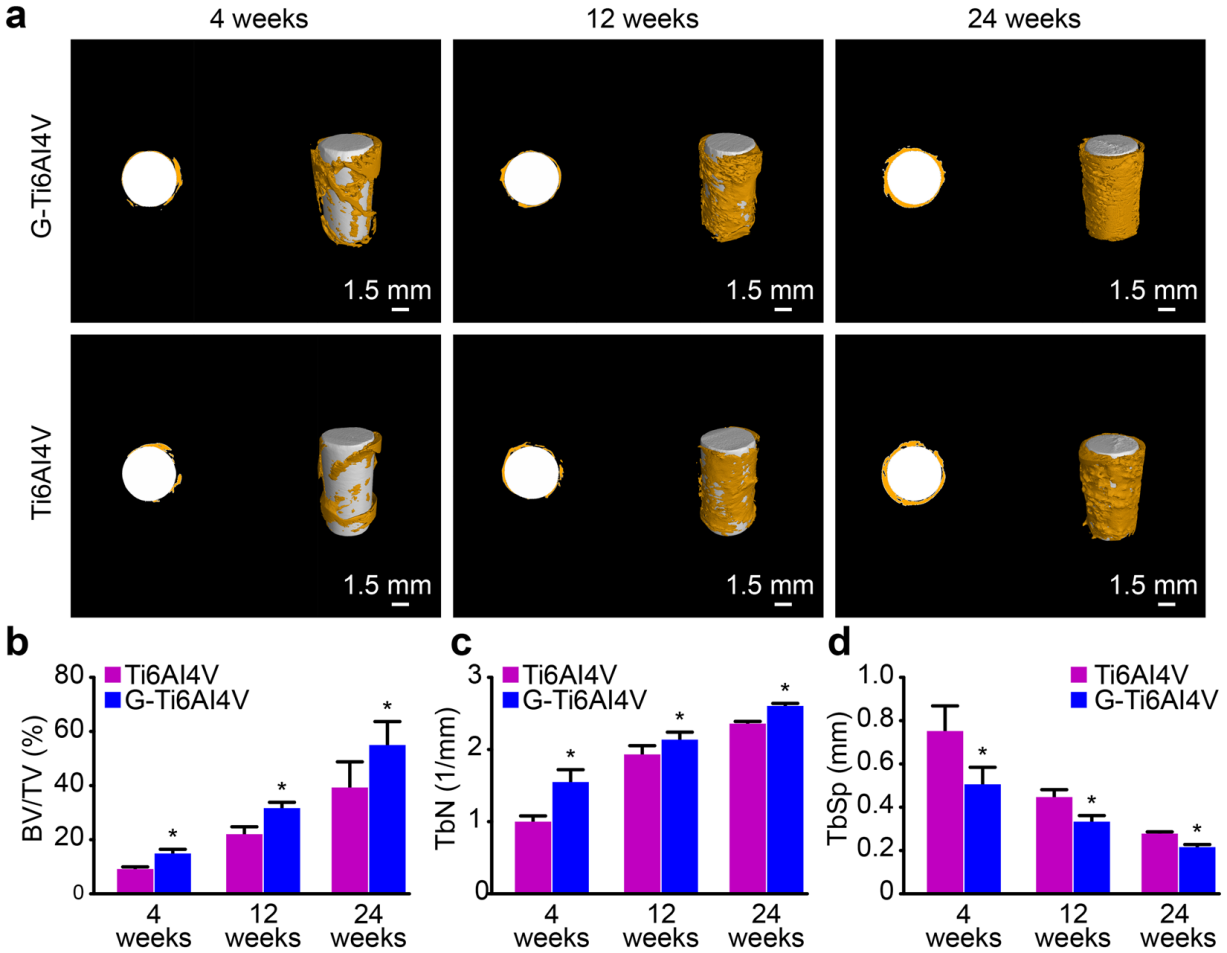
(**a**) Implanted scaffolds and new bone after 4, 12, and 24 weeks; the establishment of a unified region of interest and subsequent image reconstruction were performed through Micro-CT; yellow represents new bone, and white represents the implant. Scale: 1.5 mm. (**b**) Bone volume fraction (BV/TV) of the two groups obtained from analysis of the Micro-CT data (n = 6). (**c**) Trabecular number (TbN) and (**d**) trabecular spacing (Tb.Sp) (n = 6). The results are expressed as the mean ± SD; *represents a significant difference (*P* < 0.05).

Micro-CT was used to analyze the formation and integration status of new bone. A unified region of interest was drawn to reconstruct an image via Micro-CT. The parameters reconstructed via Micro-CT are presented in Table 1. In Fig. 3a, white represents an implanted scaffold, and yellow represents new bone tissue; the amount of new bone tissue in the two groups gradually increased over time, but at each time point, there was more bone tissue around the scaffolds in the G-Ti_6_Al_4_V group than in the Ti_6_Al_4_V group. As shown in Fig. 3b and c, the bone-volume fraction (BV/TV) and trabecular number (TbN) of the two groups increased over time, particularly from 4 weeks to 12 weeks, when BV/TV and TbN increased significantly. However, at each time point, the BV/TV and TbN obtained for the G-Ti_6_Al_4_V group were significantly higher than those of the Ti_6_Al_4_V group (*P* < 0.05). Figure 3d shows that the trabecular spacing (Tb.Sp) gradually decreased in the two groups over time, and at each time point, the Tb.Sp of the G-Ti_6_Al_4_V group was significantly lower than that of the Ti_6_Al_4_V group (*P* < 0.05).

**Table 1:** Results for Microstructural Parameters (n = 6, mean ± SD).

	4 Weeks		12 Weeks		24 Weeks	
	Ti_6_Al_4_V	G-Ti_6_Al_4_V	Ti_6_Al_4_V	G-Ti_6_Al_4_V	Ti_6_Al_4_V	G-Ti_6_Al_4_V
BV/TV (%)	8.04 ± 1.6	13.93 ± 2.33*	21.29 ± 3.25	30.89 ± 2.8*	38.52 ± 10.17	54.5 ± 9.33*
TbN (1/mm)	0.99 ± 0.1	1.54 ± 0.2*	1.93 ± 0.15	2.14 ± 0.13*	2.37 ± 0.06	2.62 ± 0.05*
Tb.Sp (mm)	0.74 ± 0.13	0.5 ± 0.09*	0.43 ± 0.04	0.32 ± 0.04*	0.26 ± 0.02	0.2 ± 0.02*

*Significant difference was found compared with the control group (*P* < 0.05).

G-Ti_6_Al_4_V indicates graphene-coated titanium alloy (Ti_6_Al_4_V). Ti_6_Al_4_V indicates non-coated titanium alloy.

BV/TV indicates the bone volume/total volume, TbN indicates the trabecular number, and Tb.Sp indicates trabecular spacing.

### Histological analysis

**Figure 4:**
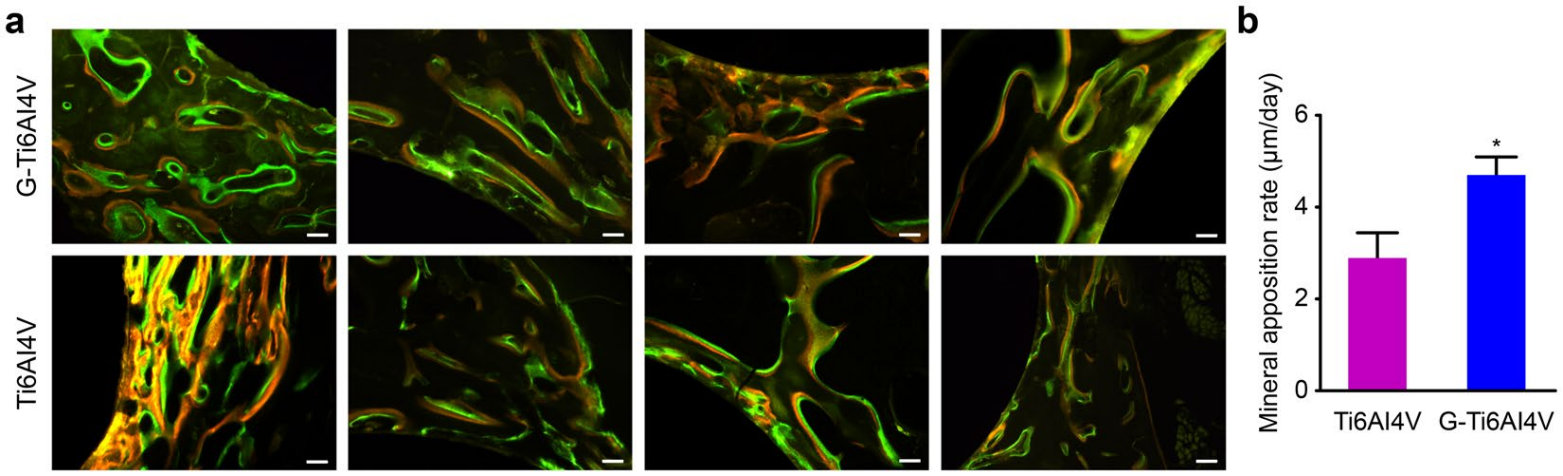
(**a**) Double-labeling immunofluorescence images of the G-Ti_6_Al_4_V group and Ti_6_Al_4_V group (Sections in the center of four scaffolds) under a fluorescence microscope. The top four images are coated titanium alloys (G-Ti6Al4V), and the bottom four are blank uncoated Ti6Al4V. After blue light irradiation, under microscopy, calcein showed green, and tetracycline showed yellow. Scale: 100 µm. (**b**) Mineral apposition rate of bone in the two groups (n = 6). The results are expressed as the mean ± SD; *represents a significant difference (*P* < 0.05).

To determine the bone mineral apposition rate (MAR), double-labeling immunofluorescence staining (Fig. 4a) was performed. The MAR was calculated as the distance between the centers of the yellow band (tetracycline) and the green band (calcein). As shown in Fig. 4b, the MAR of the G-Ti_6_Al_4_V group was 4.63 ± 0.44 µm, which was significantly higher than the value of 2.88 ± 0.55 μm for the Ti_6_Al_4_V group (n = 6, *P* < 0.05).

**Figure 5:**
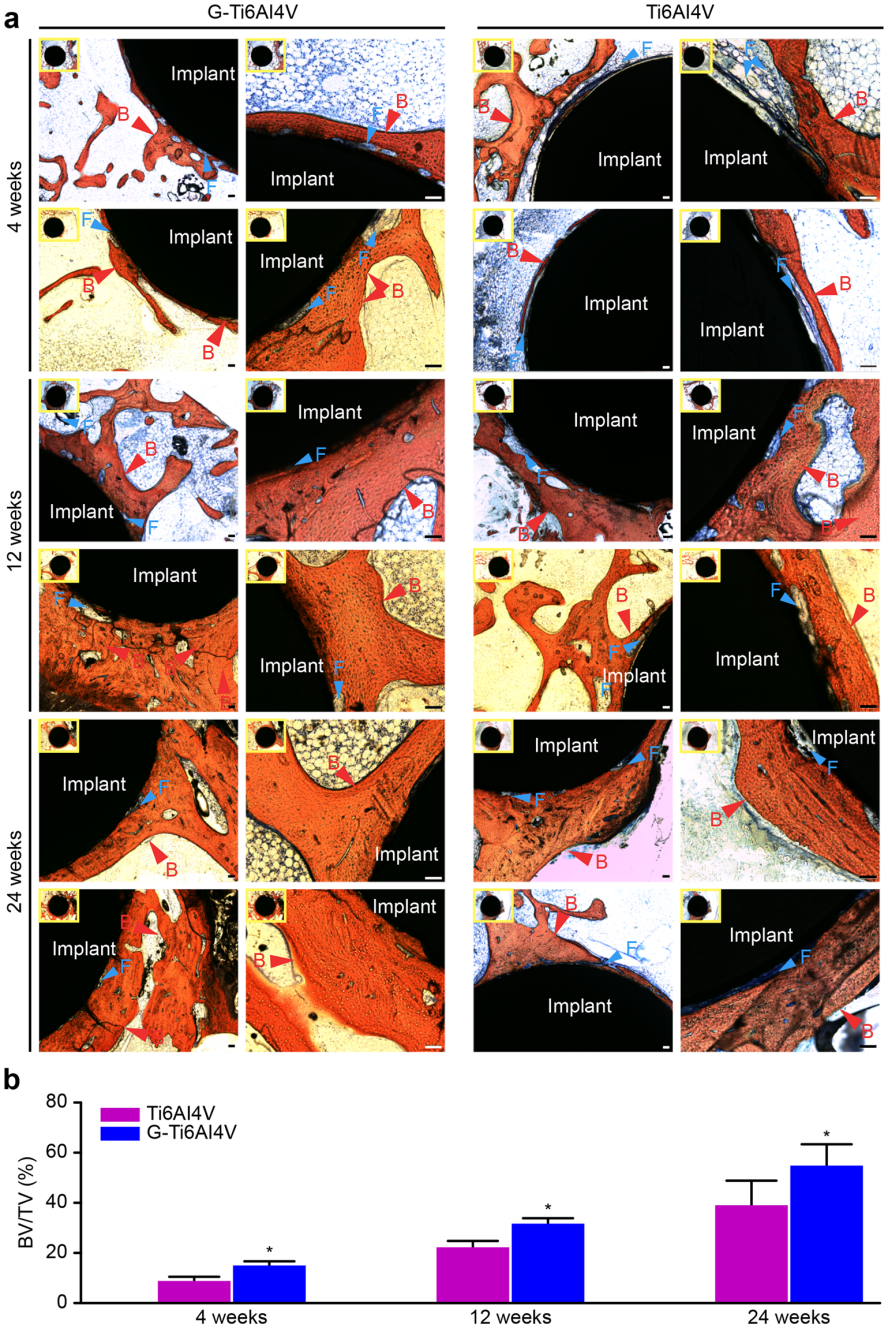
(**a**) Histological images of VG-stained hard tissue sections at 4, 12 and 24 weeks (n = 6). The implanted material (Implant) is stained black. New bone tissue (B) is stained red. Fibrous tissue (F) is stained blue. Scale: 100 µm. (**b**) New bone volume fraction/total bone volume (BV/TV) of the two groups was analyzed using Image-Pro Plus 6 software (n = 6).

Bone regeneration and osteointegration in the G-Ti_6_Al_4_V and Ti_6_Al_4_V groups were analyzed via Van Gieson (VG) staining. Figure 5 shows the implanted scaffold stained black, the bone tissue stained red, and the fibrous tissue stained blue. The degree of bone regeneration was quantified using IPP 6.0 software. At 4 weeks after surgery, we observed that more new bone formed around the G-Ti_6_Al_4_V scaffold than around the Ti_6_Al_4_V scaffold, but more fibrous tissue surrounded the Ti_6_Al_4_V scaffold. The new bone volume fraction (BV/TV, %) of the G-Ti_6_Al_4_V group (12.273 ± 1.418%) was significantly higher than that of the Ti_6_Al_4_V group (9.354 ± 1.178%, *P* < 0.05). At 12 weeks, more new bone had formed for both the G-Ti_6_Al_4_V and Ti_6_Al_4_V scaffolds, but the density and volume of new bone in the G-Ti_6_Al_4_V group were significantly higher than those in the Ti_6_Al_4_V group. Additionally, compared with the G-Ti_6_Al_4_V group, the Ti_6_Al_4_V group showed more blue-stained fibrous tissues between the implant and the bone tissue. The BV/TV in the G-Ti_6_Al_4_V group (33.548 ± 2.678%) was significantly higher than that in Ti_6_Al_4_V group (24.911 ± 2.898%, *P* < 0.05). At 24 weeks after implantation, the BV/TV of the G-Ti_6_Al_4_V group (60.164 ± 12.723%) was significantly higher than that of the Ti_6_Al_4_V group (43.868 ± 10.873%, *P* < 0.05). Moreover, the G-Ti_6_Al_4_V scaffold and bone tissue showed a firm junction, with almost no visible gap or fibrous tissue, whereas more gaps and abundant fibrous tissue were observed between the Ti_6_Al_4_V scaffold and bone tissue.

## Discussion

An ideal implant in orthopedic surgery should be accepted by the body and should be able to mimic the geometric structures and biological functions of bone. Titanium alloys are widely used in hip and knee joint replacements, fracture lag screw fixations and pedicle screw fixations^7,33,34^. However, the osteointegration of titanium alloy implants with surrounding bone tissue is unsatisfactory. In recent years, surface modifications for increasing the bioactivity and biocompatibility of Ti-based materials have become topics of interest in biomaterial engineering reseach^3,35^. Basiaga *et al*.^3^ effectively improved the biological activity and biocompatibility of an implant by coating the implant surface to introduce bioactive substances. Graphene has unique physiochemical and structural properties, and in particular, its potential for the osteogenic induction of HMSCs^32,36^ make it a promising material for promoting the surface modification of scaffold materials in bone tissue engineering^28,37–39^. To this end, we used graphene as a nanocoating material to optimize a titanium alloy (Ti_6_Al_4_V) and to improve its surface bioactivity.

As previously reported, the large-scale synthesis of graphene films by chemical vapor deposition (CVD) can be used to coat to any foreign substrate, regardless of its shape or material composition^40,41^. Studies have reported that films grown via CVD on copper substrates are predominantly composed of single-layer graphene, with a small percentage (less than 5%) of the area having multiple layers, and they are continuous across copper surface steps and grain boundaries. When the substrates are coated with graphene, peaks appear at 1560−1620 cm^−1^ (G-band) and 2660−2700 cm^−1^ (2D band)^42^. In this study, we performed Raman spectroscopy for six random sampling points on the graphene-coated Ti_6_Al_4_V scaffold and observed G and 2D peaks, as shown by the nattier blue peak (G-Ti_6_Al_4_V after 1 h of sonication), red peak (G-Ti_6_Al_4_V) and deep blue peak (G-Cu) marked with arrows in Fig. 1c. In contrast, these two peaks were not observed for the uncoated Ti_6_Al_4_V scaffolds. These results indicate that we successfully coated graphene onto the Ti_6_Al_4_V scaffolds. The ultrasonication test results suggest a stable attachment between the Ti_6_Al_4_V substrates and the graphene coating, which is important for establishing an effective implant.

*In vivo* experiments allow for more sensitive and direct analyses of the biological toxicity of materials and their effects on tissues. Graphene coatings have been reported to enhance the biological activity and biocompatibility of implants and biological scaffolds. A study by Podila *et al*.^43^ showed that graphene modification enhances the blood compatibility and biocompatibility of nickel titanium alloy scaffolds. Kim *et al*.^44^ found that graphene coatings enhance the anti-fatigue properties of carbon nanotubes. However, the effects of graphene as a coating on the osteogenic rate and volumetric growth of new bone tissue have not been studied. In our study, a rabbit femoral condyle lacunar defect model was established for the biological evaluation of the implantation of a graphene-coated Ti_6_Al_4_V scaffold in bone and its effects on osteogenesis and osteointegration. As previously reported, a push-out experiment was performed to evaluate the biomechanical properties of the bone-implant interface^45^, which is considered a representative, practical and important mechanical method for evaluating the degree of osteointegration of an implant with the surrounding bone tissue^46^. This study shows that a greater push-out strength is required to separate a G-Ti_6_Al_4_V scaffold from the femoral condyle at each time point (Fig. 2), indicating that a graphene coating significantly enhances the binding force between the implant material and the bone tissue. We also used Micro-CT to analyze the new bone volume and trabecular bone density surrounding the implant materials. The reconstruction of ROIs showed more bone trabeculae around G-Ti_6_Al_4_V scaffolds than around Ti_6_Al_4_V scaffolds (Fig. 3a). As shown in Fig. 3b and c, higher BV/TV and TbN values were obtained over time after implanting G-Ti_6_Al_4_V scaffolds than were obtained after implanting Ti_6_Al_4_V scaffolds. Moreover, VG staining of histological sections showed that new bone was actively formed around G-Ti_6_Al_4_V scaffolds and demonstrated that the presence of graphene had a positive influence on the regeneration and osteointegration of bone tissue.

The MAR represents the mineralization rate of bone tissue and can reflect the metabolism of bone tissue and the activity of osteoblasts *in vivo*^47,48^. Our histological observations of strong tetracycline labeling (yellow bands) and calcein labeling (green bands) around the scaffolds showed that the MAR of the G-Ti_6_Al_4_V group was significantly higher than that of the Ti_6_Al_4_V group (Fig. 4). These results indicate that a graphene coating improves the metabolic rate at bone defect sites and further suggests that G-Ti_6_Al_4_V scaffolds could promote faster regeneration of bone tissue at bone defects.

Cell differentiation is a critical factor for bone regeneration. Numerous studies have revealed that graphene enhances the osteogenic differentiation of cells^32,36,49–51^. We hypothesize that graphene can promote the osteogenic differentiation of cells to stimulate and enhance the biological function of osteogenesis-related factors, thus further accelerating new bone formation and osseointegration around implanted scaffolds. In our study, VG staining (Fig. 5) shows that the volume of new bone mass and the density of trabecular bone around the G-Ti_6_Al_4_V scaffold were significantly higher than were observed around the Ti_6_Al_4_V scaffold at different time points. In fact, more blue-stained fibrous tissues can be observed between the Ti_6_Al_4_V scaffold and bone tissue at each time point. In particular, after 24 weeks, the G-Ti_6_Al_4_V scaffold and bone tissue showed a firm junction, with almost no visible gap or fibrous tissue, whereas a gap and abundant fibrous tissue were observed between the Ti_6_Al_4_V scaffold and bone tissue. This result corresponds with the Micro-CT results, revealing the stimulating effect of graphene coatings on osteogenesis and osteointegration.

However, the pathway and the specific mechanism through which graphene promotes osteogenesis and osteointegration remain unknown. In subsequent experiments, we may focus on the relevant molecular mechanisms. Moreover, in future studies, we will establish large-animal models of material implantation after fracture, focus on graphene-related mechanisms during bone repair and reconstruction, obtain more data and further verify the biocompatibility and osteogenesis-inducing ability of graphene-coated materials, as well as further explore their potential applications in bone tissue engineering.

## Conclusions

We successfully prepared a stable graphene coating on a titanium alloy by means of chemical etching and physical adsorption. The graphene coating enhanced the biocompatibility of the titanium alloy scaffolds. Osteogenesis and osteointegration at the implant-bone interface were promoted, and the scaffolds further accelerated bone defect repair. In summary, graphene might become a promising material for nano-coating and bone graft substitution in clinical practice.

## Materials

### Preparation of graphene coatings

The specifications of the titanium alloy Ti_6_Al_4_V (Shenyang, China) used in this study are as follows: Ti_6_Al_4_V rods (*Φ* = 5 mm, *L* = 10 mm). Graphene films on copper substrates with 100% coverage were prepared using chemical vapor deposition (CVD; Changzhou, China). Briefly, 7% polymethyl methacrylate (PMMA) was evenly coated on the surface of a graphene-coated copper substrate (G-Cu). PMMA-graphene films were then obtained by etching Cu with an FeCl_3_ solution and were transferred to the Ti_6_Al_4_V surfaces through physical adsorption. In addition, the PMMA was then completely dissolved with acetone to obtain the graphene-coated Ti_6_Al_4_V alloy (G-Ti_6_Al_4_V).

### Stability of the coating

We used an ultrasonic cleaner (SB-5200DTD, Ningbo, China) to assess the strength and stability of the graphene coating. The power setting was 200 W at 40 kHz, and the samples were ultrasonicated for 1 h.

### Scanning electron microscopy

Scanning electron microscopy (SEM, HITACHI-S4800, Japan) at a voltage of 5 kV was performed to image the coatings, and the changes in the surface morphology of the materials and the coating coverage areas were observed.

### Raman spectroscopy

The fine structures of graphene on G-Cu, G-Ti_6_Al_4_V and Ti_6_Al_4_V (n = 5) were characterized via Raman spectroscopy (HORIBA JOBIN YVON HR800, Japan) using a laser at a wavelength of 630 nm.

### Implantation procedure

All animals were provided by the animal center of the Fourth Military Medical University. Animal studies were conducted in strict accordance with the guidelines for the Animal Management Committee of the Fourth Military Medical University and Chinese Animal Research and Management Committee.

**Figure 6:**
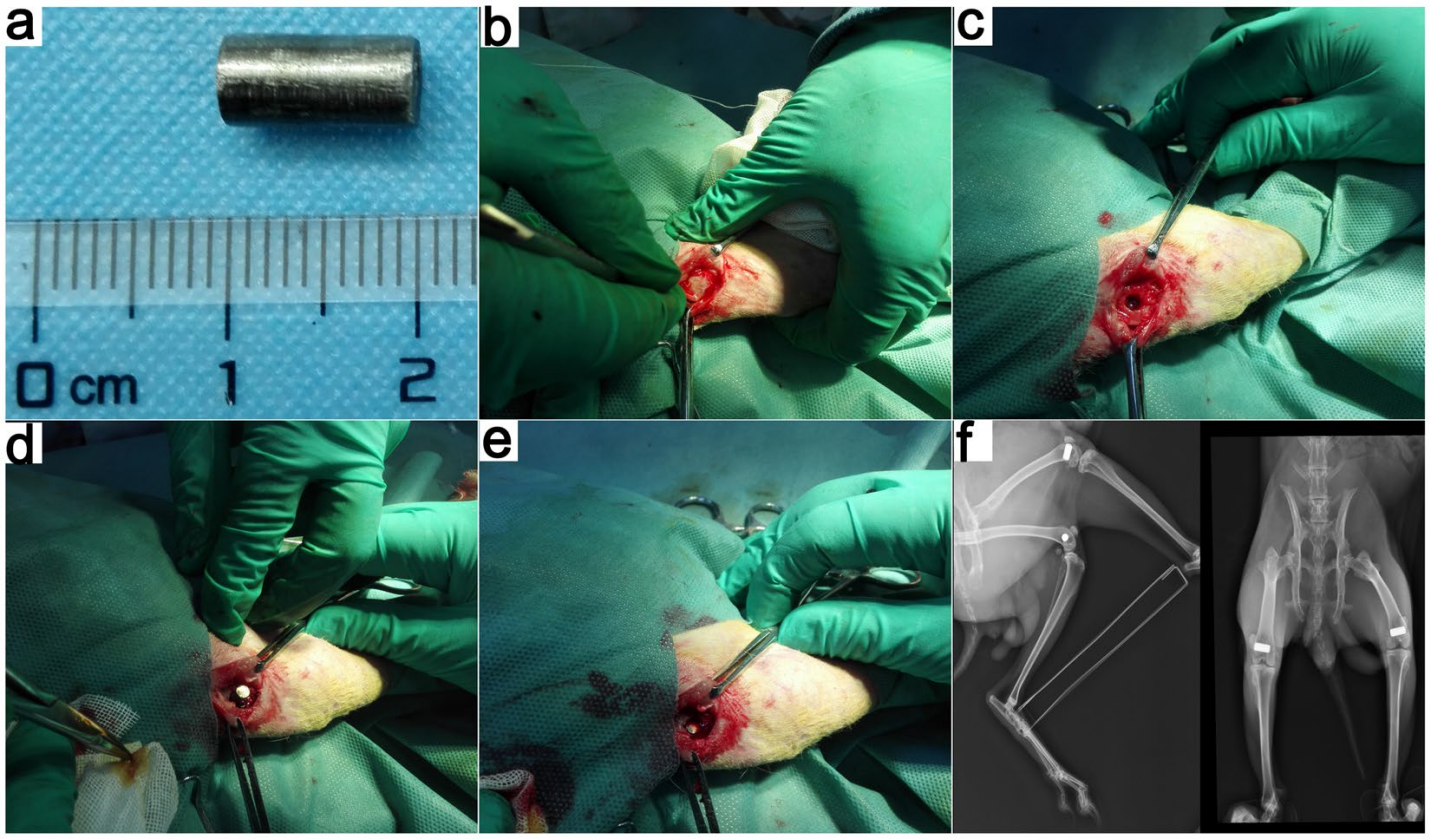
(**a**) Material specifications. (**b**–**e**) Demonstration of the surgical procedure. (**g**) X-ray images after the operation.

The G-Ti_6_Al_4_V group was the experimental group, and the Ti_6_Al_4_V group was the control group. All samples were sterilized by Co^60^ radiation (irradiation dose: 20 kGy). Thirty-six male New Zealand white rabbits, with an average weight of 3.3 ± 0.42 kg, were used (n = 6 in each group). As shown in Fig. 6, two different scaffolds were implanted into the femoral condyles of each rabbit. Briefly, 4 mg/kg xylazine hydrochloride (Shengda, China) and 30 mg/kg 2% pentobarbital sodium (Sigma, USA) were provided as an anesthetic via intramuscular injection prior to the surgery. After the commencement of anesthesia, routine entoiodine disinfection was used. To expose the femoral condyle of each rabbit, we sequentially opened the skin, exposed the subcutaneous region and dissected the surrounding fascia to expose the bone. After removing the periosteum, a bone tunnel (*Φ* = 5 mm, *L* > 10 mm) was created in the center of the lateral femoral condyle using a drill (*Φ* = 5 mm) held perpendicular to the longitudinal axis of the femur. A G-Ti_6_Al_4_V rod was implanted in the tunnel, and the terminus was closed with bone wax. The wound was cleaned and sutured. Using the same procedure, a Ti_6_Al_4_V rod was placed in the other femoral condyle of the same rabbit. Three days after surgery, each rabbit was administered gentamicin (5 mg/kg) and penicillin (50 kU/kg). At 4, 12 and 24 weeks after surgery, there were no signs of infection in any of the rabbits. At each time point, six rabbits were sacrificed for the relevant analyses. The rabbits were sacrificed 14 d before the injection of tetracycline (80 mg/kg, Sigma) and 4 d before the intramuscular injection of calcein (8 mg/kg, Sigma) for double immunofluorescence staining detection (n = 6).

### Biomechanical testing

Six rabbits were randomly chosen for sacrifice at 4, 12 and 24 weeks, and 12 samples were obtained for biomechanical testing (push-out test). Soft tissues attached to bones were removed prior to testing, and the metal surfaces of the implanted materials were exposed. The samples were tested using a universal mechanical testing machine (MTS-858 Mini Bionix, USA). A special fixture was used to hold the test samples in place and was placed on the base of the universal mechanical testing machine. Thimbles were used (*Φ* = 4 mm) at a loading rate of 1 mm/min to gradually separate the implant and femoral condyle, and the maximum failure load was recorded (push-out force).

### Micro-computed tomography

Six rabbits were randomly chosen for sacrifice 4, 12 and 24 weeks after surgery, and the femoral condyles were then carefully dissected and fixed in 80% ethanol for 2 weeks. Detection was performed through micro-computed tomography (Micro-CT; Cheetah Y.; Yxlon, Hamburg, Germany). The X-ray source voltage was set to 80 kV, the beam current was 200 mA, the scanning angular rotation was 360°, and the angular increment was 0.40°. For each specimen, a 5 × 10 mm^2^ cylindrical area was selected as the region of interest, and the region from the middle portion of the bone tunnel along its longitudinal axis was reconstructed and analyzed using VG-Studio MAX 2.0 software (Graphics Heidelberg, Volume, Germany). The bone volume/total volume fraction (BV/TV, %), trabecular number (TbN, 1/mm) and trabecular spacing (Tb.Sp, mm) were measured.

### Histological analysis

After Micro-CT analysis, the samples were dehydrated with an alcohol gradient (70–100%) and soaked in a methyl methacrylate (MMA) solution for 3 weeks. A hard-tissue microtome (Leica Microtome, Wetzlar, Germany) was then used to slice the hard tissues at a slice thickness of 200 µm. Each hard-tissue slice was placed on a glass slide and polished to obtain 50-µm-thick samples. Tetracycline and calcein fluorescence emissions were observed through fluorescence microscopy (Nikon, Japan). After blue-light irradiation under a microscope (emission filter, ET 420 nm LP; excitation filter, AT 350/50 × nm.), calcein appeared green, and tetracycline appeared yellow. The distance between two markers (µm) was measured using Image-Pro Plus 6.0 (IPP) software, and the mineral apposition rate (MAR, m/d) of new bone was calculated. All of the slices were then stained with Van Gieson (VG) stain, and the formation and integration of the new bone were observed by optical microscopy (DM6000B, Microsystems Leica, Germany). Osteogenesis was qualitatively analyzed on the basis of VG-stained pathological sections using IPP 6.0 software.

### Statistical analyses

Quantitative data were analyzed using SPSS 22.0 (Chicago, USA) and Graph Pad Prism 6.0 (CA, USA). At least three experimental samples were collected for each test. The statistical methods used in this study included t-tests and analysis of variance. The mean ± SD were calculated, and *P* < 0.05 was considered statistically significant.
